# Perinatal Food Deprivation Modifies the Caloric Restriction Response in Adult Mice Through Sirt1

**DOI:** 10.3389/fphys.2021.769444

**Published:** 2021-12-02

**Authors:** Isaac Peña-Villalobos, Fabiola A. Otárola, Bárbara S. Casas, Pablo Sabat, Verónica Palma

**Affiliations:** ^1^Department of Ecological Sciences, Faculty of Sciences, Universidad de Chile, Santiago, Chile; ^2^Laboratory of Stem Cells and Developmental Biology, Faculty of Sciences, Universidad de Chile, Santiago, Chile; ^3^Center of Applied Ecology and Sustainability (CAPES), Santiago, Chile

**Keywords:** epigenetic, fetal programming, gluconeogenesis, life history trait, maternal gestational weight gain, PPARs, predictive adaptive hypothesis, Sirt1

## Abstract

Variations in the availability of nutritional resources in animals can trigger reversible adjustments, which in the short term are manifested as behavioral and physiological changes. Several of these responses are mediated by Sirt1, which acts as an energy status sensor governing a global genetic program to cope with changes in nutritional status. Growing evidence suggests a key role of the response of the perinatal environment to caloric restriction in the setup of physiological responses in adulthood. The existence of adaptive predictive responses has been proposed, which suggests that early nutrition could establish metabolic capacities suitable for future food-scarce environments. We evaluated how perinatal food deprivation and maternal gestational weight gain impact the transcriptional, physiological, and behavioral responses in mice, when acclimated to caloric restriction in adulthood. Our results show a strong predictive capacity of maternal weight and gestational weight gain, in the expression of *Sirt1* and its downstream targets in the brain and liver, mitochondrial enzymatic activity in skeletal muscle, and exploratory behavior in offspring. We also observed differential responses of both lactation and gestational food restriction on gene expression, thermogenesis, organ masses, and behavior, in response to adult caloric restriction. We conclude that the early nutritional state could determine the magnitude of responses to food scarcity later in adulthood, mediated by the pivotal metabolic sensor Sirt1. Our results suggest that maternal gestational weight gain could be an important life history trait and could be used to predict features that improve the invasive capacity or adjustment to seasonal food scarcity of the offspring.

## Introduction

Food access for animals may be limited by spatial or temporal resource availability (e.g., seasonal or environmental stochasticity). These variations in the availability of nutritional resources in animals can trigger reversible adjustments, which in the short term are expressed behaviorally and physiologically (Grover et al., 2007; Harris et al., 2010; Rimbach et al., 2014). Among mammalian species, including humans, such responses to food deprivation or caloric restriction (CR) typically involve a reduction in the mass of organs with high metabolic output, a decrease in behavioral activity, and changes in the expression of genes related to both catabolism and anabolism (Overton and Williams, 2004; Most et al., 2017). Rodents display several particularities, in terms of physiological outputs and allocation of energy, such as reduction of body temperature, an increase of exploratory behavior, and increase of intestine length (Peña-Villalobos et al., 2019, 2020).

An evolutionarily conserved protein, known as Sirtuin 1 (Sirt1), has been proposed as a key factor in the response to CR, acting as an energy status sensor through its NAD^+^-dependent deacetylase activity. Indeed, Sirt1 controls a global genetic program to cope with changes in the nutritional status. Absence of Sirt1 results in a metabolically inefficient animal that fails to adapt to CR conditions (Boily et al., 2008). Sirt1 acts in most tissues with important metabolic and neuroendocrine functions (e.g., liver, skeletal muscle, and hypothalamus). For example, in the hepatic tissue, Sirt1 activates gene expression related to gluconeogenesis and inhibition of adipogenesis, promotes fat mobilization, and stimulates brown adipose tissue remodeling (see Li, 2013; Ye et al., 2017). In the hypothalamus, levels of Sirt1 have been related to exploratory responses and appetite control (Libert et al., 2011; Robinette et al., 2020), whereas in the intestine Sirt1 cooperates to foster the expansion of gut adult stem cells during caloric restriction (Igarashi and Guarente, 2016). Hence, this protein senses and controls several processes in different tissues, based on its context-dependent expression and activation (Chen et al., 2012).

The role of Sirt1 as a master regulator of metabolism occurs mainly through two mechanisms. Its deacetylation activity has been reported acting both on non-histone proteins and histones. Sirt1 actively deacetylates proteins involved in signal transduction or gene transcription. For example, Sirt1 deacetylates PGC-1α, a potent coactivator of a plethora of transcription factors (such as peroxisome-proliferator-activated receptors or PPARs) impacting whole-body energy expenditure (see Feige and Auwerx, 2007; Rodgers et al., 2008). Sirt1 is also widely recognized as a crucial epigenetic regulator modifying chromatin structure by histone deacetylation, as described in several pathological conditions (Lee and Kemper, 2010; Rifaï et al., 2018) and in perinatal nutrition (Botden et al., 2012; Suter et al., 2012).

There is growing evidence demonstrating a key role of the perinatal environment in the setup of physiological responses in adult individuals when subjected to CR during development. Studies in rodents (*Mus musculus* and *Rattus* sp.) indicate that the reduction in energy intake during pregnancy and lactation produces persistent effects in the progeny during adulthood under *ad libitum* feeding conditions, such as altered proteomic profile, reductions in insulin and leptin sensitivity, modifications in hypothalamic function, hyperphagia, reduction in thermogenesis, and abnormalities in body mass, among others (Garcia et al., 2010; Aravidou et al., 2016; Ramírez-López et al., 2016; Agnoux et al., 2018).

These studies suggest that in the face of perinatal CR, metabolic programming can occur, which would ensure the ability to acquire energy in the postnatal environment through the expression of a “thrifty phenotype” (Hales and Barker, 2001). An extension of the so-called thrifty phenotype hypothesis is the adaptive predictive responses hypothesis (Bateson et al., 2014). This hypothesis suggests that individuals under perinatal nutritional deprivation exhibit physiological adjustments (i.e., programming or fetal epigenetic programming, for a review, see Zhu et al., 2019), which would confer metabolic capacities suitable for a food-scarce environment that animals may face later. Despite the key role of Sirt1 in metabolism, behavior, and epigenetic function, there are no studies to date that evaluate the implication of Sirt1 in the development of such predictive adaptive responses.

To gather evidence suggesting that Sirt1 is a mediator of predictive adaptive responses, here, we evaluated the effect of perinatal food deprivation (i.e., restriction in pregnancy and lactation) and maternal body condition (maternal gestational weight gain) on physiological (i.e., organ size, thermogenic capacity, subcutaneous temperature, mitochondrial activity in muscle, Sirt deacetylase activity in the liver, and expression of Sirt1 downstream genes in the liver, brain, and brown adipose tissue) and behavioral features (exploratory activity in an open field test) in adult rodents (Male *Mus musculus*, BALB/c) acclimated to both *ad libitum* feeding and CR in adulthood. Our results suggest that Sirt1 is involved directly in the CR-response strategy playing a central role in metabolic changes setting the ground for further studies on its potential long-lasting epigenetic effects on offspring.

## Materials and Methods

### Animal Acclimation

Eighteen adults BALB/c strain of *Mus musculus* females (3 months old) were obtained from the central animal housing facilities at the Faculty of Sciences, Universidad de Chile. These females were bred in pairs with nine males (3 months old) in the same conditions. Pregnancy was identified through a vaginal plug, and the females were assigned to three conditions: *ad libitum* feeding (AL, *n*=6), CR in pregnancy (RP, *n*=6) with 70% of normal feed and CR in lactation (RL, *n*=6) with 70% of normal feed. For the latter two treatments, we estimated the daily food consumption previously, in an exploratory study on other females from the same origin, strain, and age. Specifically, we observed that during pregnancy (embryonic days E10-E19) and lactation (postnatal days P0-P23), females eat 7.86±3.0 and 13.77±3.28 g of dry food per day (Prolab RMH 3000, Labdiet, United States), respectively. Then, despite the progressive increment in food intake during pregnancy and lactation, we determine to provide 70% of the mean value (i.e., 5.50 and 9.64 g, a 30% of caloric restriction), implying a greater energetic challenge, in females with larger litters, late pregnancy, and after the first week of weaning. To compensate for the energetic effort of lactation between mothers, we regrouped pups in litters of eight individuals per mother in all treatments. We measured the body mass of all females, before pregnancy and one day before birth, on day 18 of pregnancy. Additionally, we measured the body mass of the offspring weekly from birth to the end of the experiment.

**Figure 1:**
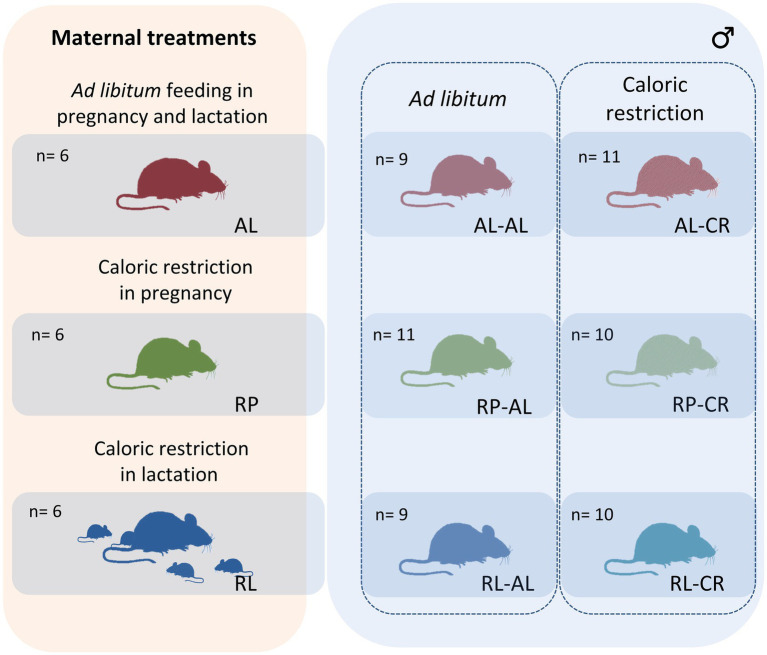
Experimental design. We performed three maternal feeding conditions: control (*ad libitum* or AL), caloric restriction in pregnancy (RP), and caloric restriction in lactation (RL). The adult male offspring of every maternal treatment were split in two groups: *ad libitum* access to food (AL) or twenty days of caloric restriction (CR). In the right panel, abbreviations refer to maternal condition (AL, RL, and RP) and in the left panel, additional adult treatment (AL or CR).

After lactation (i.e., day 25 after birth), offspring were housed individually in standard cages with paper flakes and kept in a temperature-controlled room, at 25° ± 1°C, in a LD=12:12cycle, with water and food *ad libitum* (Prolab RMH 3000, Labdiet, United States) for three months, until adulthood. Then, they were separated into six groups of 9 to 11 animals, with no siblings in the same group (Figure 1). In this experiment, we only use males because females of the BALB/c strain of *Mus musculus* rise the maximal size at an asymptotic level at a different time than males, investing energy in growth for more time.

For each maternal condition, in the half of the adult individuals, we restricted the food intake by 40% of the *ad lib* food intake, i.e., we provided 0.6^*^ 3.61 g of dried food (Prolab RMH 3000, Labdiet, United States) for 20days. This commercial food was employed during their growth, in lactation and pregnancy. Besides, the colony of mice was bred for at least three generations with this food. All animal procedures were in accordance with the Chilean legislation and were approved by the Institutional Animal Care and Use Committees at the Universidad de Chile (Certificate no: 17068-FCS-UCH) and Comisión Nacional de Investigación Científica y Tecnológica (CONICYT, now called ANID).

### Body Temperature

Body temperature during the experiments was measured by means of a subcutaneous transponder (IPTT-300, BioMedic Data Systems, Seaford, DE), with an accuracy of ±0.2°C in the calibrated range of 32–43°C, as well as a handheld reader (DAS-6006/7 Smart Probe, Bio Medic Data Systems Inc.). At the age of two months, the transponders were implanted subcutaneously in the lower back of animals using a sterilized needle assembly provided by the manufacturer (BioMedic, Seaford, DE), under anesthesia (isoflurane + oxygen). Temperature was measured during acclimation in the light phase.

### Exploratory Behavior

The exploratory behavior was assessed through an open field test both at the beginning and end of the acclimation period in 58 individuals (we lost two recordings). Tests were performed in a square acrylic box (thickness of 2 mm) of white color, with an internal surface of 1 m^2^ and a height of 30 cm. These measurements were recorded within the activity phase of the animals (9 pm to 2 am) as briefly described: at the beginning of the measurement, the rodents were placed in the center of the field and for 10 min, they were filmed in darkness using an infrared camera (Wanscam HW0026) located 2 meters above the exploration field, in articulated support. The camera was connected to a computer, in an adjoining room where filming was recorded. Between each session, the soil surface was completely cleaned with 70% ethanol. Running or walking is considered as an exploration index, therefore, as a proxy of exploration or trajectory, we measured the number of squares crossed in the open field (internal surface of 1m^2^ was digitally sectioned in a grid of 25 squares of 0.2×0.2 m). Video analysis was performed with *Phobos* software.

### Organ Mass

After all measurements, animals were sacrificed by cervical dislocation, and inner organs were weighed (± 0.001 g; Analytical Balance, AUX Series, Shimadzu Scientific Instruments) for all individuals, *n*=60 (see Supplementary Table 1). The intestine was extracted, and its content was gently removed mechanically, followed by the recording of intestine mass and length (± 0.001 g and 0.1 cm, respectively). Immediately after sacrifice, the musculature present in the hind legs (*biceps femoris, gracilis, semitendinosus, rectus femoris, anterior tibialis, and vastus lateralis*) was dissected on ice. The tissues were stored at −80°C, pending the completion of enzymatic assays. Brain, liver fraction, and brown adipose tissue (BAT) were conserved in RNAlater (Thermo Fisher) at −80°C, for further extraction of RNA.

### Enzymatic Activity

The enzymatic activity determinations were carried out using hind legs muscles homogenates of six individuals per treatment. Samples were homogenized on ice (1:10 w/v) in 0.1 M phosphate buffer supplemented with 2 mm EDTA (pH 7.3) using an Ultra Turrax homogenizer (20,000 rpm). The samples were then sonicated at 130 W using an Ultrasonic Processor VCX 130 on ice 14 times in 20-s cycles with 10-s intervals between cycles. Homogenates were then centrifuged at 15,000 rpm for 15 min at 4°C to obtain a post-mitochondrial fraction. The supernatant was transferred into a new tube, to avoid transferring the upper lipid layer present in the homogenates. Protein concentration was determined using the Bradford method, with bovine serum albumin as the standard. We measured the activity of two mitochondrial enzymes: cytochrome c oxidase (E.C. 1.9.3.1) and citrate synthase (E.C. 4.1.3.7). An increase in the activity of these enzymes likely reflects changes in both the functional properties and the density of mitochondria. The cytochrome c oxidase (COX) activity was quantified using a microplate spectrophotometric method. Enzyme activity was determined in a reaction mixture containing 10 mm Tris–HCl (pH 7), 120 mm KCl, 250 mm sucrose, and cytochrome c reduced with dithiothreitol in a final volume of 0.2 ml. The decrease in extinction at 550 nm was monitored in a Thermo Scientific Multiskan GO UV/VIS spectrophotometer at 25°C. Enzyme activity was calculated using an extinction coefficient of 21.84 mm^−1^ cm^−1^ at 550 nm for cytochrome c. The citrate synthase (CS) activity was measured as follows: The enzyme assay medium contained 10 mm Tris–HCl (pH 8.0), 10 mm 5,5’dithiobis- (2 nitrobenzoic acid), 30 mm acetyl Coenzyme A (acetyl CoA), and 10 mm oxaloacetic acid (OAA) in a final volume of 0.2 ml; these reagents were omitted in controls. Citrate synthase catalyzes the reaction between acetyl CoA and OAA to form citric acid. The increase in extinction at 412 nm was measured in a Thermo Scientific Multiskan GO at 25°C. Enzyme activity was calculated using an extinction coefficient of 13.6 mm^−1^ cm^−1^ at 412 nm. All enzyme activities are reported as specific activity per gram of protein (μmol min ^−1^ mg protein^−1^).

We analyzed the deacetylase activity in nuclear protein extracts from liver tissues of three individuals per treatment. For the isolation and extraction of nuclear proteins from liver tissues, we used an EpiQuik Nuclear Extraction Kit (EpiGentek, United States) and measured enzymatic activity by mean of Epigenase Universal SIRT Activity/Inhibition Assay Kit (EpiGentek, United States), following the manufacturer’s protocol.

### Gene Expression

Total RNA was obtained from brain, liver, and BAT from five individuals per treatment, by phenol-chloroform extraction using Trizol. cDNA was synthesized using 1 μg of RNA and a M-MLV reverse transcription kit, employing a DNAse treatment. Relative expression of several genes related to *Sirt1* was assessed by qPCR (Agilent Technologies Thermocycler, Santa Clara, CA, United States) using specifically designed primers as indicated in Supplementary Table 2. Data were analyzed by calculating the expression fold change *via* 2-ΔΔCt, and gene expression was normalized to that of tissue specific reference/housekeeping genes for CR, as suggest by Gong et al. (2016) (*Gapdh, B2m, Hmbs*, and *Hprt*, see Supplementary Table 2).

### Statistical Analysis

The averages of the morphological, biochemical, and behavioral variables between treatments were compared through nested and factorial ANOVA, using perinatal and adult feeding as factors. In the cases in which the variables correlated with body mass, the residuals of each variable against body mass were used from the regression analyses.

To associate levels in *Sirt1* related gene expression and phenotypic changes to the effect of maternal feeding, we performed Pearson correlations between physiological variables and relative gene expression. To identify differential relationships between inner fat deposits, organ mass, and body mass changes during CR, we evaluated the relationship of these variables by mean Pearson correlation, for each treatment. We evaluated the association between physiological traits of offspring and variables related to maternal weight (one day before birth, at day 18 of pregnancy) or maternal gestational weight gain (delta between final and initial body mass), using linear regression analysis.

To evaluate the effect of maternal mass in gene expression and offspring body mass, a path analysis using AMOS (SPSS) was performed. For that, we computed path coefficients among measured variables to test the overall path diagram of the observed data. We used maximum likelihood to estimate parameters. The significance of each parameter is presented only in significant cases, and the relative magnitude of regression weights was reported in color key (edited in Microsoft Publisher).

## Results

Sirt1 mediates effects of CR in mammals, as widely demonstrated in the literature (see Guarente and Picard, 2005; Boily et al., 2008). Several lines of evidence indicate an elaborate set of physiological adaptations to caloric intake mediated by Sirt1. Nevertheless, the idea that Sirt1 could be an active mediator of predictive adaptive responses has not been explored so far. Hence, here we set out to answer the following question: Does perinatal CR promote the development of adaptive responses to food scarcity that are correlated with *Sirt1* expression?

### Organ Masses Are Affected Differentially by Perinatal Conditions

Maternal gestational weight gain was not affected by the experimental treatment [ANOVA *F*_(2, 57)_=0.709, *p*=0.496] indicating that the ranges of variation were similarly (mean; 9.28 g; min=4.30 g; max=14.10 g; SD=2.68, Supplementary Table 1). This variation could be influenced for variables, such as the number of embryos, as both variables’ trend to show a positive correlation (*p*=0.072), with a range of 2–9 litter size.

**Figure 2:**
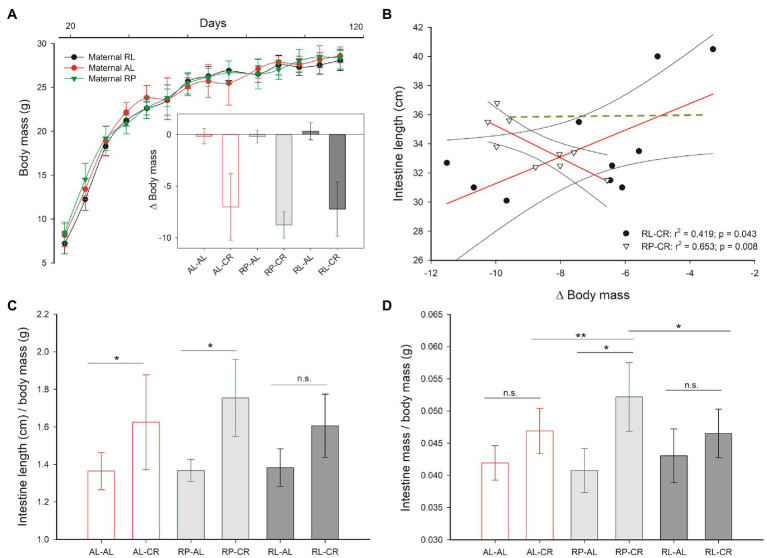
**(A)** Body mass increment under three perinatal feeding conditions and body mass change, under adult CR versus *ad libitum* control. **(B)** Correlation analysis between body mass changes and intestine length for RP-CR and RL-CR groups (all other treatments in green, *p*>0.05). **(C,D)** Detailed analysis of intestine length and mass of offspring after perinatal and adult treatments as indicated. One asterisk: *p*<0.05; two asterisks: *p*=0.05.

Maternal feeding regime affected offspring body mass throughout growth up to 60th days of age, with greater body mass in PR individuals, in relation to RL and lower mass in female mice [main effects ANOVA *F*_(12, 32)_=2.357, *p*=0.026 for treatments, and *F*_(6, 16)_=11.401, *p*<0.001, for sex]. Nevertheless, maternal feeding regime did not modify the mass reduction of offspring during CR in adulthood (Figure 2A; see Supplementary Table 1).

Despite the overall effects on body mass, the perinatal feed modified the organ mass response to caloric restriction in adulthood. As shown in Supplementary Table 1, the nested analysis indicated that inner organs of adult mice were differentially affected by caloric restriction depending on their perinatal conditions. Thus, for example, epididymal fat mass was not reduced when animals were exposed to RP and intestine mass was diminished significantly in RL animals.

In order to understand the relationship between organs mass changes and body mass, we analyzed the association among these variables. Data showed a greater intestinal mass in animals displaying a higher body mass reduction in the RP-CR group. Otherwise, this relationship was inverse in the RL-CR group (Figure 2B) and absent in control (AL-AL). Hence, prenatal CR generates a differential response to adult CR resulting in individuals with heavier and longer intestines (Figures 2C,D).

### Gene Expression in Liver, Brain, and BAT Is Affected and Predicted by the Perinatal Condition

**Figure 3:**
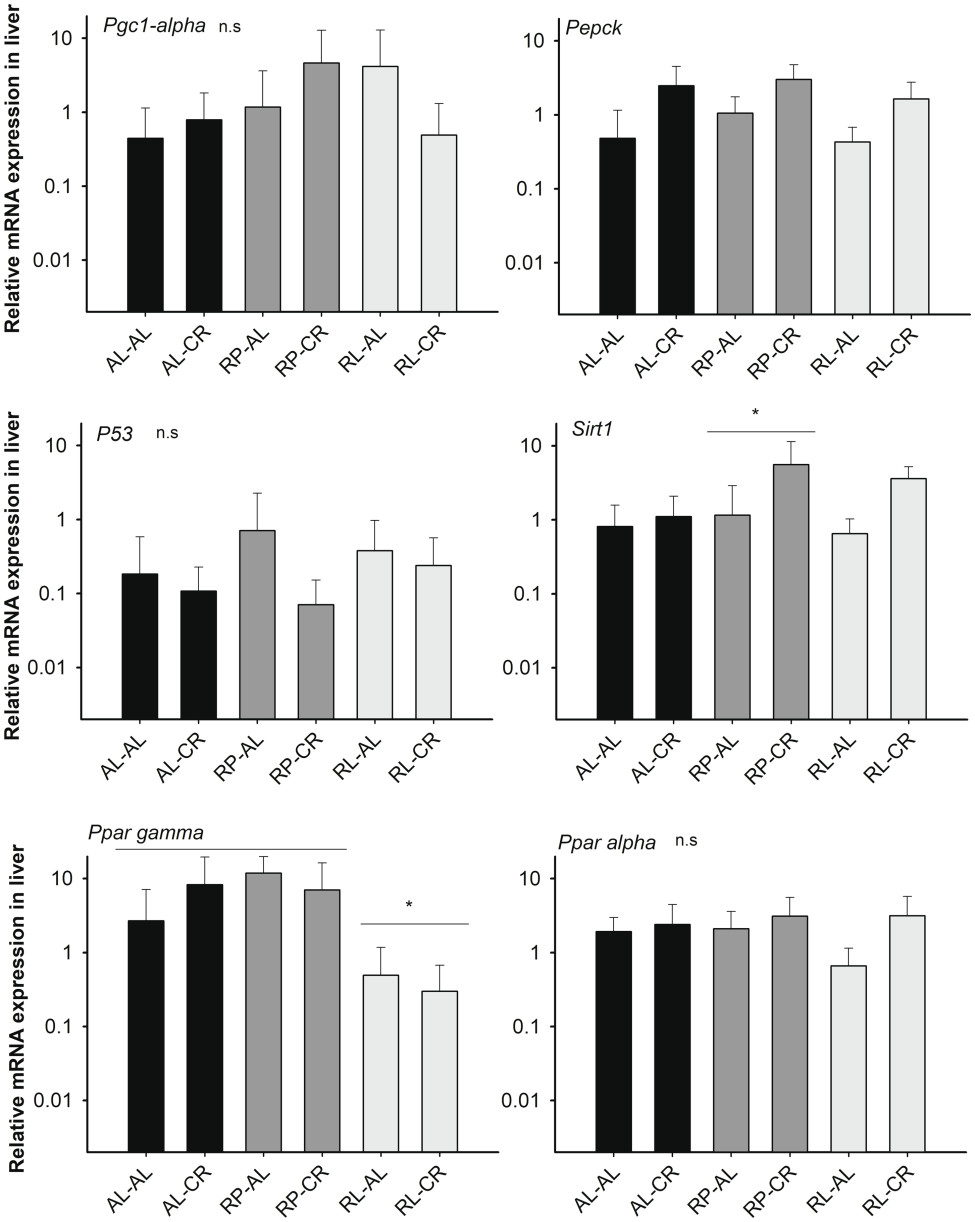
Relative expression of *Sirt1* (respect to *B2m*)-related genes in the liver of mice under treatments of perinatal and adult food availability as indicated. See statistical results in main text for all graphs. One asterisk: *p*<0.05.

In pooled data, *Sirt1* was upregulated in the liver in animals under CR [*F*_(3, 25)_=3.592, *p*=0.028] but only showed statistical difference in the RP treatment group between adult control and the CR condition (Fisher LSD test). In a similar fashion, rodents under CR had higher *Pepck* expression, using *B2m* and *Hmbs* as housekeeping genes [*F*_(3, 25)=_ 5.349, *p*=0.006; *F*_(3, 25)_=3.842, *p*=0.022, respectively]. Interestingly, *Ppar gamma* expression was affected by the perinatal condition, with a lower expression found in RL animals compared to AL and RP groups [*F*_(2,25)_=4.251, *p*=0.026; see also non-significant results in Figure 3].

**Figure 4:**
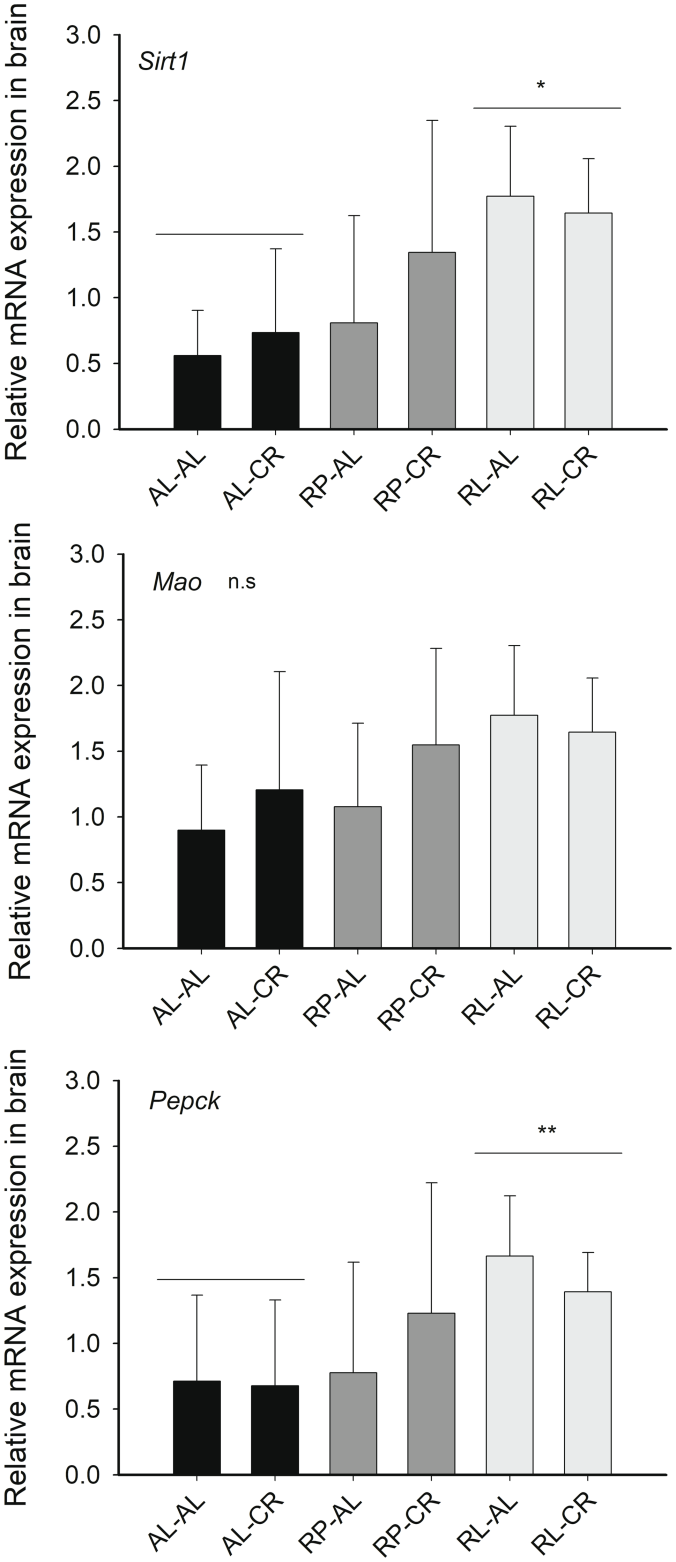
Relative expression of genes related with *Sirt1* (respect to *B2m*) in the brains of mice from six groups of perinatal and adult food availability. See statistical results in main text for all graphs. One asterisk: *p*<0.05; two asterisks: *p*=0.07.

We found a significant effect by the maternal food regime on *Sirt1* expression in the brain (Figure 4), ~2.6-fold higher in RL animals compared to AL treatment, with RP animals expressing intermediate values [Factorial ANOVA: *F*_(2, 23)_=5.933, *p*=0.008]. We observed a similar trend in *Pepck* expression [*F*_(2, 22)=_ 2.993, *p*=0.071].

**Figure 5:**
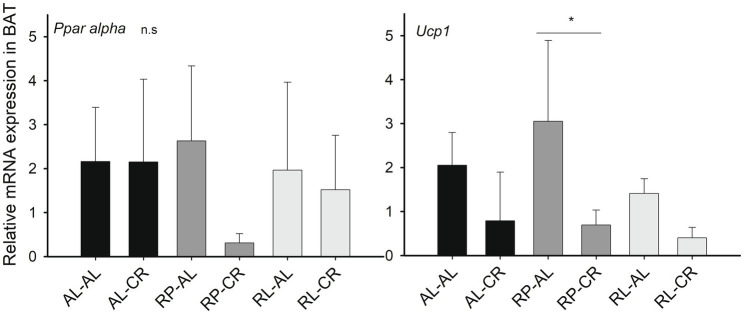
Relative expression levels of *Sirt1*-related genes (respect to *Hprt*) in the BAT of mice under treatments of perinatal and adult food availability as indicated. See statistical results in main text for all graphs. One asterisk: *p*<0.05.

When analyzing the thermogenic capacity, we found a 2.7-fold reduction in *Ucp1* expression under CR in BAT [Factorial ANOVA: *F*_(1, 23) =_ 17.313, *p*<0.001]. We also observed that the RP-AL group had a significantly higher expression of *Ucp1* compared to RP-CR [*F*_(3, 23)_=7.884, p<0.001, Figure 5].

**Figure 6:**
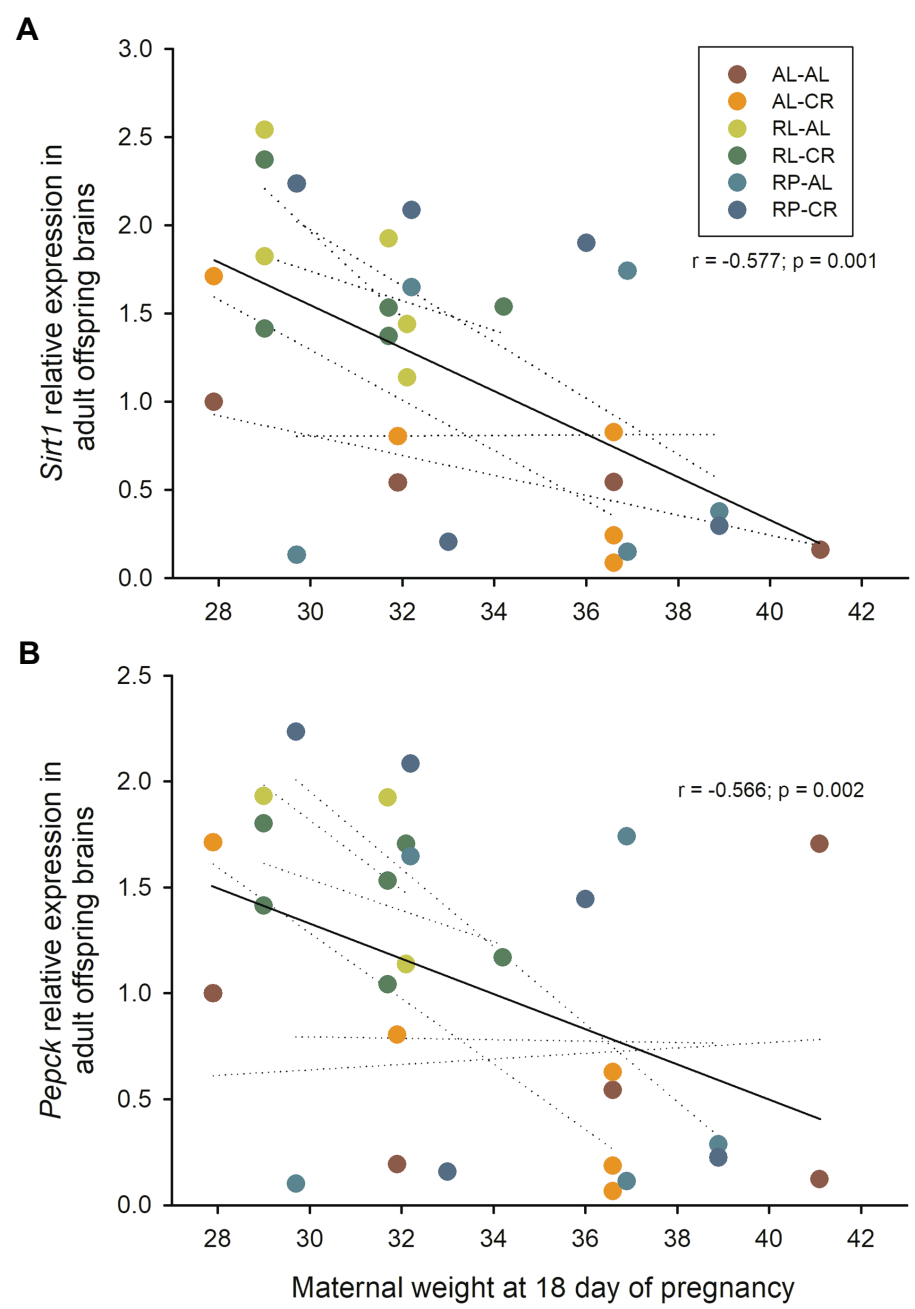
**(A)** Relationship between Sirt1 relative expression (respect to B2m) in the brains of adult male BALB/c mice, and the body weight of their mothers at the end of pregnancy. **(B)** Relationship between Pepck relative expression (respect to B2m) and maternal body weight. Dashed line: linear regression of each treatment.

Interestingly, we found a strong association between maternal mass during pregnancy and gene expression when all data were pooled. Brain *Sirt1* and *Pepck* were negatively associated with maternal gestational weight gain, explaining ~24% of the variance in expression levels of the gene (*r*=−0.491; *p*=0.007 and *r*=−0.483; *p*=0.011, respectively). Expression levels of *Sirt1* and *Pepck* were also negatively associated with the mother’s body mass at day 18 of pregnancy, explaining ~30% of the variability (*r*=−0.577; *p*=0.001; *r*=−0.566; *p*=0.002, respectively, Figure 6). Maternal weight was also correlated with *Mao* expression in the brain (*r*=−0.399; *p*=0.032; *r*=−0.394; *p*=0.035; for *Gapdh* and *B2m*, respectively). In the liver, *p53* expression was negatively correlated with maternal body mass (*r*=−0.412; *p*=0.033), and *Ppar alpha* showed a statistical trend when associated with maternal gestational weight gain (*r*=0.386; *p*=0.032) while *Ppar gamma* with mass at day 18 of pregnancy (*r*=0.402; *p*=0.025).

To analyze the independent effect of perinatal treatment on Sirt1 expression, we accounted for the maternal weight effect by performing an ANCOVA using maternal gestational weight gain and maternal weight at birth as covariates. This analysis revealed a significant effect of treatments [*F*_(2, 25)_=4.45, *p*=0.022 and F_(2, 25)_=3.63, *p*=0.041, respectively]. In consequence, variation in Sirt1 mRNA levels is affected by maternal body weight and perinatal treatments.

We did not measure specific activity of Sirt in tissues; however, we assayed the total deacetylase activity from nuclear proteins in the liver. No differences were found in deacetylase activity between adult treatments in the AL maternal condition *p*=0.601; in RP *p*=0.795 or the RL group *p*=0.196. Activity in the RP-AL group was strongly associated with *Pepck* (*r*=0.992; *p*=0.008).

### Catabolic Activity in Skeletal Muscle Is Predicted by Maternal Weight

Regarding catabolic activity in skeletal muscle, we found a strong effect of perinatal feeding on the CS activity between experimental treatments. Specifically, the RP group had higher activity than the AL and RL groups (*F*_(2, 33)_=8.607, *p*<0.001). RP-AL and RP-CR have significantly greater CS activity than AL-CR. COX activity was 2.5-fold higher in RP than in RL animals (F_(2, 33)=_7.752, *p*=0.002).

**Figure 7:**
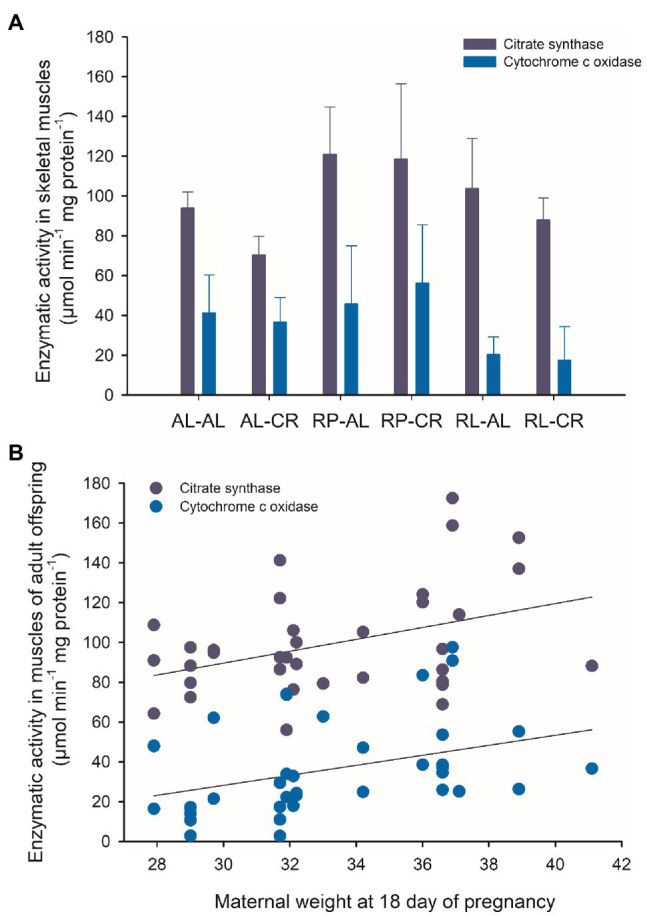
**(A)** Enzymatic activity of citrate synthase and cytochrome c oxidase from skeletal muscle of adult male BALB/c mice, from six treatments of perinatal and adult food availability. **(B)** Relationship between offspring enzymatic activity and body weight of their mothers at the end of pregnancy. See statistical results in main text.

Maternal weight at day 18 of pregnancy explained ~14% of the variability of metabolic activity of skeletal muscle. The analysis showed a positive association between maternal weight and both CS and COX values (*r*=0.393; *p*=0.018; *r*=0.378; *p*=0.023, respectively, Figure 7). Additionally, we observed a differential relationship between the metabolic activity of skeletal muscle and Sirt activity in the liver. The RP-AL treatment showed a positive association between citrate synthase activity and deacetylase activity, instead RL-AL treatment showed a negative correlation (*r*=0.997; *p*=0.047; *r*=−0.951; *p*=0.049, respectively). When all data were pooled, a negative association between COX activity and deacetylase activity was found in the brain (*r* =−0.469; *p*=0.032).

### Exploratory Behavior Is Correlated With Maternal Gestational Weight Gain

**Figure 8:**
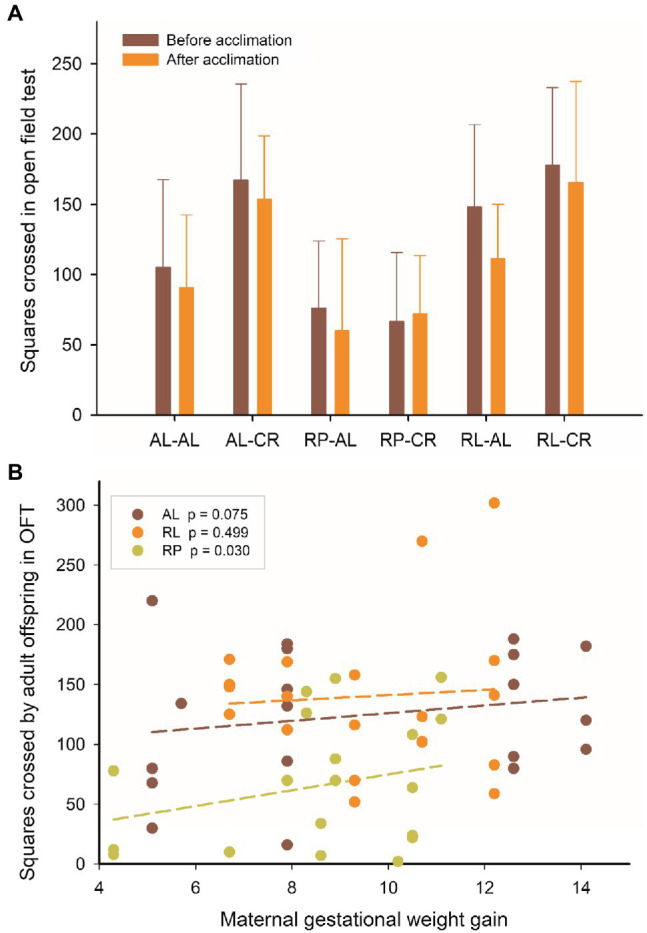
**(A)** Squares crossed in the open field test before and after acclimation, in adult male BALB/c mice, from six groups of perinatal and adult food availability. **(B)** Pooled data of squares crossed in the open field test (before acclimation to adult CR) and the body weight gain of their mothers at the end of pregnancy. See statistical results in main text.

The open field tests revealed an effect of maternal and adult feeding on the number of squares crossed (before and after adult acclimation to CR). The RP group had half of the exploratory activity as AL and RL [Repeated measures ANOVA, *F*_(4, 102)_ =8.064, *p* <0.001]. Of note, we found that the number of squares crossed before acclimation is correlated with maternal gestational weight gain in the RP group (*r* =0.487; *p* =0.030), but not in either the RL or AL (*r* =−0.165; *p* =0.499; *r* =0.418; *p* =0.075, respectively, Figure 8).

There was a significant relationship between *Sirt1* gene expression in the brain and the number of squares crossed in open field test in RL-CR group (*r*=0.999; *p*=0.034). There was also a positive relationship between *Mao* expression in the brain and the times that animals entered the central area of the open field, in the RL-AL group (*r*=0.998; *p*=0.039) and in the pooled RL group (*r*=0.645; *p*=0.044).

### Thermogenic Activity Is Reduced in Animals With Early Food Deprivation

**Figure 9:**
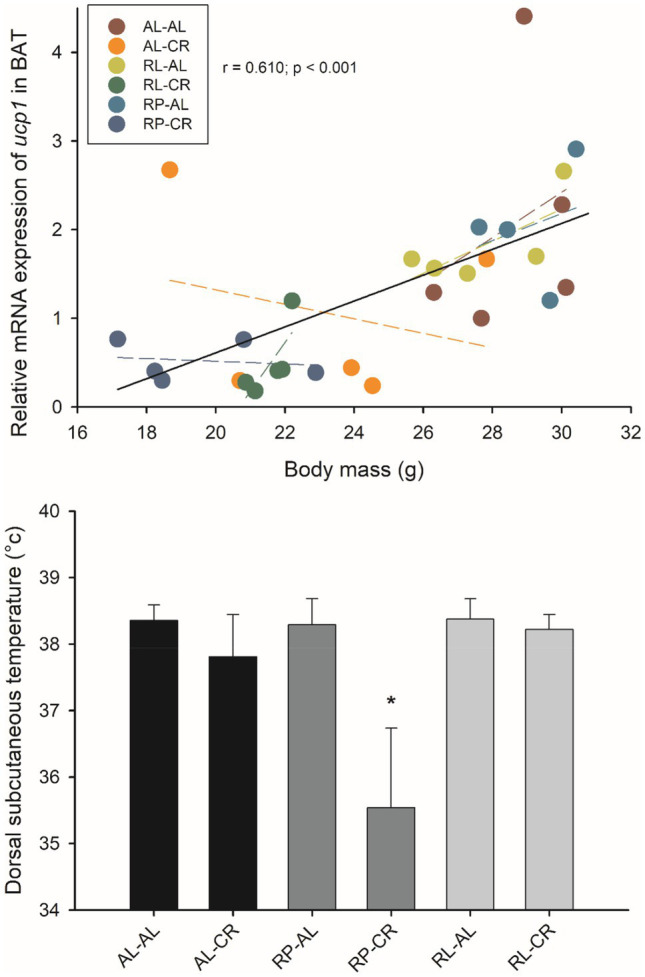
Relationship between *Ucp1* in BAT and body mass in adult male BALB/c mice, from six groups of perinatal and adult food availability. Dashed line: linear regression of each treatment. Inner graphs show subcutaneous temperature among treatments. One asterisk: *p*<0.05.

A positive relationship between Δ mass and Δ temperature (before and after acclimation) could be established when all data were pooled (*r*=0.410; *p*=0.006). There was a negative association between body mass and temperature in the RL-CR group (*r*=−0.739; *p*=0.015). Dorsal subcutaneous temperature decreased in the RP-CR group [*F*_(5, 50)_=31.735, *p*<0.001] at the end of acclimation; averaging 35.5°C in these animals (Figure 9).

*Ucp1* expression increased ~2.7-fold in the BAT of AL when subjected to CR [*F*_(3, 23)_=5.864, *p*=0.004]. We observed a positive association between *Ucp1* and body mass and temperature (*r*=0.500; *p*=0.007; *r*=0.433; *p*<0.021, respectively). These results are consistent with using both *Gapdh* and *Hprt* as the housekeeping gene for both body mass and temperature parameters (*r*=0.610; p<0.001; *r*=0.654; *p*=0.002, respectively).

We found a difference in correlation between BAT mass and *Ppar gamma* in the liver. Specifically, RL and RP had a negative correlation (*r*=−0.900; *p*=0.038; *r*=−0.911; *p*=0.032, respectively) and AL had a positive correlation (*r*=0.855; *p*=0.030). When all data were pooled *Ppar alpha* had a positive relationship with body mass when using either *Hprt* and *Gapdh* as housekeeping genes (*r*=0.544; *p*=0.003; *r*=0.449; *p*=0.019, respectively). An intriguing result was observed in the RP-CR group since there was an inverse relationship between *Ppar gamma* and S*irt1* in both the liver and body temperature parameters (*r*=−0.9871; *p*=0.002; *r*=−0.994; *p*<0.001, respectively). In the RP-CR group, we observed a negative association between the intestine length and BAT mass (*r*=−0.668; *p*=0.049). A negative correlation between n-aminopeptidase activity and temperature (*r*=−0.825; *p*=0.043; *r*=−0.908; *p*=0.005, respectively) was identified between AL-CR and RL-CR groups.

### Quantification of Maternal Effects in Offspring Phenotype

**Figure 10:**
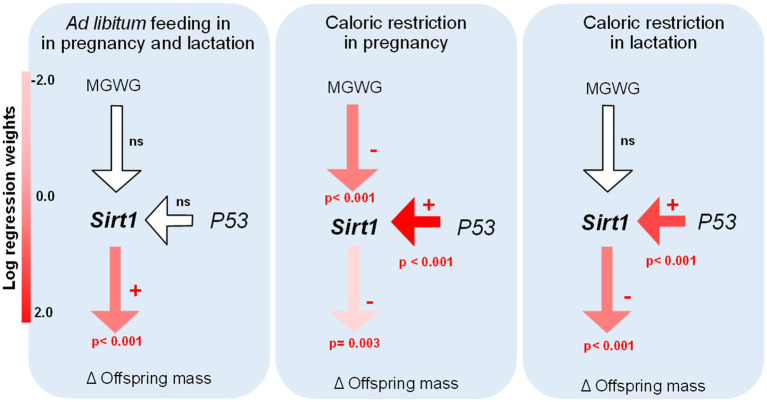
Evaluation of causal models in adult male BALB/c mice, considering all treatments of perinatal and adult food availability. Examination of the relationships between key genes in the liver (S*irt1* and *P53*, respect to *B2m*), body mass changes in adult acclimation (caloric restriction), and maternal gestational weight gain. The significance of each parameter is presented only in significant cases, and the magnitude of regression weights is represented by a color key.

Remarkably, the path analysis revealed a significant and negative effect of maternal gestational weight gain on *Sirt1* expression in the liver in the RP treatment group, suggesting that a lower increase in body mass during the pregnancy implies a higher expression of *Sirt1* in offspring. This finding, is in line with the negative association found between both variables, observed when data were pooled (*r*=−0.554; *p*=0.032) or when the treatments AL-CR and RL-CR were considered separately (*r*=−0.990; *p*=0.001; *r*=−0.866; *p*=0.058, respectively). The model showed a positive and significant relationship between *P53* and *Sirt1* expression levels in the liver, but only in perinatal restricted females (RP and RL conditions). *Sirt1* expression in the liver had a significant effect on offspring body mass in all treatment groups; however, the direction and magnitude differed between experimental groups depending on the type of perinatal feeding. In control animals acclimated to adult CR (i.e., AL-CR), we found that *Sirt1* mRNA levels were positively related with changes in body mass in adult offspring (before and after acclimation). Contrary, in perinatal restricted animals, this relationship was negative (Figure 10).

## Discussion

The main objective of our study was to evaluate the effect of perinatal feeding on features related to energy budgeting in a rodent model challenged with CR as adults and to evaluate the potential role of Sirt1 in the development of predictive adaptive responses. Our findings support the hypothesis of metabolic programing, developmental plasticity, and maternal effects in the establishment of patterns of physiological responses, determined early, and in a transgenerational way. Interestingly, *Sirt1* expression levels in offspring were strongly predicted by maternal gestational weight gain, indicating that this variable should be accorded greater attention as an important life history trait.

### Effects of Perinatal Feeding on Organ Mass Control

Some organs and fat deposit masses were differentially affected by perinatal feeding after the acclimation of offspring to CR. Maternal weight gain during pregnancy can predict the expression of *Ppar alpha* and *Ppar gamma* in the liver in a~16%. The use of nutritional fueling mediated by PPARs could promote the oxidation of nutritional reserves. Since PPARs act as nutritional sensors, our results suggest that most likely, in response to food restriction, differential rates of fatty acid catabolism, lipogenesis, and ketone body synthesis can occur (Hashimoto et al., 2000) in organs, such as liver, heart, skeletal muscle, BAT, and kidney (see Pawlak et al., 2015).

The aforementioned differences in organ mass and fat deposits suggest the development of trade-offs between lipid droplet formation in the liver and BAT mass under perinatal food restriction. There was a different association between BAT mass and *Ppar gamma* expression in the liver of offspring, depending on their perinatal condition. Specifically, under RL or RP, the mass of BAT was inversely related to *Ppar gamma* expression, and with AL treatment (i.e., control), the relationship was positive.

The only strong correlation between deacetylase activity in the liver (i.e., Sirt activity) and the expression of *Pepck* was in individuals under prenatal CR. These findings suggest an important role of perinatal food restriction in the modification of nutritional resource management. Thus, a differential metabolic predisposition to use energetic fuels or sources (from organs and adipose tissues) could be potentially determined by Sirt1 promoting acetylation of downstream targets, such as PPARs. The latter would agree with the glucose intolerance and lipid metabolic adaptations in response to intrauterine and postnatal CR documented in male adult rats (Garg et al., 2013). It was recently described that in late life stages, hepatic autophagy is altered by nutritional cues set during late fetal and early postnatal life (Devarajan et al., 2019). Further studies are needed to understand how perinatal nutritional conditions may affect the development of various organs and how this process is controlled.

### Thermogenic Effect of Perinatal Caloric Restriction, an Adaptive Response

It is well known that CR induces a thermogenic decrease in rodents (Severinsen and Munch, 1999; Wang et al., 2006; Cao et al., 2009; Almundarij et al., 2017). Here, we found that mothers subjected to CR passed the legacy of that stress exposure to their offspring. Remarkably, we observed a strong effect of CR when affecting maternal nutrition during pregnancy, but not during lactation. Specifically, we observed a significant reduction in body temperature and *Ucp1* expression in BAT in the RP-CR group. This finding is in line with previous studies in rats showing that moderate CR during gestation programs offspring for lower BAT thermogenic capacity, probably by thyroid and sympathetic signaling (Palou et al., 2015). Interestingly, the strong reduction in thermogenic capacity is evoked only under restrictive conditions, i.e., cold or CR. In summary, our results suggest an adaptive predictive response, as evidenced by the development of thrifty phenotypes in thermogenic features, that allow a decrease in the energetic cost, when individuals experience a prenatal CR.

### Exploratory Behavior and Muscular Metabolism Determined by Early Feeding

The thrifty phenotype hypothesis is the result of the epidemiological association between poor fetal or infant feeding and the subsequent development of health problems, such as metabolic syndrome, diabetes, and other pathologies (Hales and Barker, 2001; Victora et al., 2008). Here, we present new evidence and propose a thrifty phenotype as a possible adaptive feature, in the context of adult nutritional deprivation. Indeed, the RP group showed a reduced exploratory activity in OFT, and at the same time a greater enzymatic activity of CS and COX in leg muscles. This finding suggests a high catabolic activity, but a lower energetic use in locomotor behavior and thermogenic activity (this group has the lower body temperature, ~35.5). The latter supporting the idea of individuals with a lower energetic expense but not reduced machinery for ATP production. Such reduction in behavior has been reported in rats when analyzing the effects of perinatal and preconception food deprivation, in adult behavior in exploratory tests (Levay et al., 2008; Reyes-Castro et al., 2012). Interestingly, we determined that for the RP treatment group, maternal weight at day 18 and gestational weight gain predict these features, explaining 14% of the variability of metabolic activity in skeletal muscle and~24% of exploratory behavior.

### Maternal Body Weight as a Predictor of Gene Expression and Physiological Traits

Based on our data, we identified a strong predictive capacity of maternal body weight on *Sirt1* expression, and several other genetic and physiological variables, such as catabolic activity in skeletal muscle. This finding leads to several questions related to the understanding of metabolic programming. Indeed, future experiments on *Sirt1* (and its downstream signaling pathways) and mitochondrial activity (e.g., citrate synthase and cytochrome c oxidase) should consider early nutritional status, particularly during pregnancy. Based on the results of our study, metabolic programming is greatly influenced by the maternal energetic condition. These observations shed light on the importance of the predictability of physiological features from gene expression to phenotype. The predictability found in our study agrees with the employment of maternal anthropometry as a predictor of birth weight in humans (Nahar et al., 2007). Specifically, there is strong support in the literature about the influence of maternal body mass index or gestational weight gain on the development of diseases or certain phenotypes, such as preterm delivery risk, risk of obesity at any age, and expression of energy sensing genes in the placenta, such as mTOR downstream genes (Schack-Nielsen et al., 2005; Oken et al., 2008; Martino et al., 2016; Oskovi Kaplan and Ozgu-Erdinc, 2018).

The relationship between maternal mass and offspring gene expression could be the result of SIRT1 deacetylase activity on histones, acting as a molecular mediator of the fetal epigenome and maternal metabolome, as reported in non-human primates under maternal high-fat diets (Suter et al., 2012). A moderate maternal energy restriction during gestation could program a certain *Sirt1* expression profile in different peripheral tissues in mice, which in turn could be related to obesity predisposition in adulthood (Palou et al., 2013). Recent studies suggest that SIRT1 can act as an epigenetic target during fetal development accounting for susceptibility to metabolic outcomes (see Lee, 2015).

### Maternal Effects in the Regulation of Sirt1 Expression

The present study indicates that early food restriction in gestation or during lactation can modify the effect of *Sirt1* expression in the liver in relation to the body mass in offspring. Specifically, a weak and inverse relationship between both variables in animals under CR and perinatal restricted origins (i.e., an increase in *Sirt1* expression reflects a reduction in body weight) was identified. Paradoxically, in control condition, *Sirt1* expression was positively associated with an increase in body mass in CR rodents. The negative association between maternal weight gain and *Sirt1* expression was detected in the RP-CR group, with an increase of 1.95 (± 0.26) in *Sirt1* relative expression, for each gram of body weight not gained at pregnancy (with respect to the *ad libitum* condition). This finding supports the predictive adaptive responses since the presence of *Sirt1* has been considered a condition for metabolic efficiency during CR (Boily et al., 2008).

Nemoto et al., in 2004 (Nemoto et al., 2004) reported that stimulation of *Sirt1* transcription by FOXO3a is mediated through two P53-binding sites present in the *Sirt1* promoter, by a nutrient-sensitive physical interaction between FOXO3a and P53. In their experiment, *Sirt1* expression was not induced in starved P53-deficient mice. Here, we analyzed the relationship between *P53* and *Sirt1* expression, under different perinatal conditions. To the best of our knowledge, we report for first time that perinatal feeding can modulate the intensity of the relationship between *P53* and *Sirt1* expression in offspring. However, only under RL-CR and RP-CR, the level of response was significantly greater and statistically significant. These results suggest that under perinatal food restriction an increment in *P53* expression induces in turn a higher expression of *Sirt1*.

We conclude that the early nutritional state could determine the magnitude of responses to food scarcity later in life. This response could be mediated by SIRT1 in an adaptive fashion, with a high predictability by means of maternal gestational weight gain. Further investigation is needed to understand the detailed mechanism of this transgenerational plasticity, the relationship between critical time windows in development and the epigenetic mechanisms of these predictive adaptive responses. Our findings open new perspectives about the ecological and evolutionary role of thrifty phenotypes in rodents, suggesting that maternal gestational weight gain could be an important life history trait that could be used to predict features that improve invasive capacity (the factors that determine whether a species introduced becomes an invader) and promote physiological and behavioral adjustments to seasonal food scarcity of offspring.

## Data Availability Statement

The original contributions presented in the study are included in the article/Supplementary Material, further inquiries can be directed to the corresponding author.

## Ethics Statement

The animal study was reviewed and approved by Institutional Animal Care and Use Committees at the Universidad de Chile (Certificate no: 17068-FCS-UCH) and Comisión Nacional de Investigación Científica y Tecnológica (CONICYT, now called ANID).

## Author Contributions

IP-V, VP, and PS: conceptualization, writing-original draft, and writing-review and editing. IP-V, FO, and BC: investigation. IP-V: formal analysis. All authors contributed to the article and approved the submitted version.

## Funding

This work was supported by the Fondo Nacional de Desarrollo Científico y Tecnológico Postdoctoral ANID FONDECYT Postdoctoral: 3180108 to IP-V; ANID PIA/BASAL FB0002 and CONICYT/ANID Fellowships for PhD # 21150781 (BC).

## Conflict of Interest

The authors declare that the research was conducted in the absence of any commercial or financial relationships that could be construed as a potential conflict of interest.

## Publisher’s Note

All claims expressed in this article are solely those of the authors and do not necessarily represent those of their affiliated organizations, or those of the publisher, the editors and the reviewers. Any product that may be evaluated in this article, or claim that may be made by its manufacturer, is not guaranteed or endorsed by the publisher.
