# Supplementary material for: Perinatal Food Deprivation Modifies the Caloric Restriction Response in Adult Mice Through Sirt1
Source: Front Physiol. 2021 Dec 2;12:769444. doi: 10.3389/fphys.2021.769444 (PMC8675943; doi:10.3389/fphys.2021.769444)
Supplement: Supplementary file 1 [file Table_1.DOCX]

Supplementary Material

Table 1: Cohort study data indicating maternal gestational weight gain and body mass of mice receiving three different perinatal feeding treatments. Additionally, this table shows distinctive features of their offspring in adulthood, subdivided into two treatments of food availability. Letters indicate Tukey test differences. Abbreviations, AL: Alimentation *ad libitum*; CR: caloric restriction; RP: caloric restriction in pregnancy; RL: caloric restriction in lactation; MGWG: maternal gestational weight gain; BAT: brown adipose tissue.

|  | Perinatal feeding *ad libitum* (AL) | | | | Caloric restriction in pregnancy | | | | Caloric restriction in lactation | | | |  |  |
| --- | --- | --- | --- | --- | --- | --- | --- | --- | --- | --- | --- | --- | --- | --- |
| Variables | AL-AL | | AL-CR | | RP-AL | | RP-CR | | RL-AL | | RL-CR | | F | p-value |
| MGWG (g) | 9.46 ±3.87 |  | 9.8±3.45 |  | 8.54±2.46 |  | 8.92±1.95 |  | 9.21±2.13 |  | 9.79±2.17 |  | 0.35 | 0.88 |
| Mother body mass (g) | 33.67 ±5.39 |  | 34.13±4.62 |  | 34.55±3.20 |  | 34.45±3.06 |  | 33.11±3.75 |  | 33.92±3.98 |  | 0.17 | 0.97 |
| Body mass change (g) | -0.16±0.73 | ^a^ | -7.00±3.23 | ^b^ | -0.19±0.61 | ^a^ | -8.75±1.28 | ^b^ | 0.32±0.84 | ^a^ | -7.21±2.62 | ^b^ | 75.65 | < 0.0001 |
| Body mass (g) | 28.24±1.64 | ^a^ | 22.25±3.13 | ^b^ | 28.12±1.10 | ^a^ | 19.47±1.73 | ^b^ | 28.22±1.73 | ^a^ | 21.18±2.24 | ^b^ | 59.25 | < 0.0001 |
| Heart (g) | 0.19±0.02 | ^a^ | 0.15±0.03 | ^b,c^ | 0.17±0.02 | ^a,c^ | 0.13±0.02 | ^b^ | 0.17±0.013 | ^a,c^ | 0.14±0.03 | ^b,c^ | 13.52 | < 0.0001 |
| Liver (g) | 1.25±0.37 | ^a^ | 1.08±0.25 | ^a,b,c^ | 1.30±0.17 | ^a^ | 0.75±0.13 | ^b,c^ | 1.28±0.31 | ^a^ | 0.98±0.27 | ^a,b,c^ | 9.93 | < 0.0001 |
| Intestine (g) | 1.19±0.11 | ^a,c^ | 1.04±0.11 | ^b,c^ | 1.15±0.13 | ^a,c^ | 1.02±0.14 | ^b,c^ | 1.21±0.13 | ^a^ | 0.98±0.11 | ^b^ | 9.49 | < 0.0001 |
| Intestine (cm) | 38.46±2.56 | ^a^ | 35.51±2.78 | ^a,b^ | 38.42±1.41 | ^a^ | 33.87±1.75 | ^b^ | 38.93±2.51 | ^a^ | 33.83±3.71 | ^b^ | 13.08 | < 0.0001 |
| Spleen (g) | 0.13±0.02 | ^a,d^ | 0.07±0.03 | ^a,f^ | 0.15±0.08 | ^c,d^ | 0.05±0.01 | ^b,f^ | 0.12±0.01 | ^a,d^ | 0.07±0.03 | ^a,f^ | 14.60 | < 0.0001 |
| Epididymal fat (g) | 0.54±0.09 | ^a^ | 0.13±0.16 | ^b^ | 0.40±0.11 | ^a ,c^ | 0.19±0.12 | ^b,c^ | 0.52±0.17 | ^a^ | 0.07±0.13 | ^b^ | 29.70 | < 0.0001 |
| BAT (g) | 0.19±0.03 | ^a,c^ | 0.13±0.05 | ^a^ | 0.19±0.04 | ^c^ | 0.10±0.03 | ^b^ | 0.18±0.03 | ^a,c^ | 0.11±0.05 | ^b^ | 14.60 | < 0.0001 |
| Cecum (g) | 0.22±0.02 | ^b^ | 0.18±0.03 | ^a^ | 0.26±0.04 | ^b^ | 0.17±0.03 | ^c^ | 0.22±0.05 | ^b^ | 0.19±0.06 | ^a,c^ | 9.61 | < 0.0001 |
| Kidney (g) | 0.48±0.05 | ^a^ | 0.40±0.19 | ^a,b^ | 0.49±0.04 | ^c^ | 0.30±0.04 | ^b^ | 0.46±0.04 | ^a,b^ | 0.35±0.05 | ^b^ | 10.08 | < 0.0001 |
| Reproductive (g) | 0.79±0.15 | ^a^ | 0.58±0.11 | ^b^ | 0.74±0.18 | ^a^ | 0.50±0.09 | ^b^ | 0.78±0.06 | ^a^ | 0.64±0.09 | ^a,b^ | 13.07 | < 0.0001 |
| Stomach (g) | 0.20±0.07 |  | 0.24±0.04 |  | 0.20±0.02 |  | 0.23±0.04 |  | 0.19±0.02 |  | 0.24±0.04 |  | 1.31 | 0.28 |
| Brain (g) | 0.47±0.02 | ^a,c^ | 0.47±0.02 | ^a,c^ | 0.46±0.02 | ^a,c^ | 0.44±0.02 | ^b^ | 0.47±0.02 | ^c^ | 0.45±0.02 | ^a,c^ | 3.15 | 0.03 |

Table 2: qPCR Primer sequences for mouse gene expression assays.

| **Gen** | **Sequence** | |
| --- | --- | --- |
| *sirt1* | Fwd | CAGACCCTCAAGCCATGTTT |
|  | Rev | ATTCCTGCAACCTGCTCCAA |
| *pparα* | Fwd | CCAGTACTGCCGTTTTCACA |
|  | Rev | TTGAGGTCTGCAGTTTCCGA |
| *pparg* | Fwd | AGGGCGATCTTGACAGGAAA |
|  | Rev | ATCGAAACTGGCACCCTTGA |
| *pgc-1* | Fwd | AATGCAGCGGTCTTAGCACT |
|  | Rev | AGGGTCATCGTTTGTGGTCA |
| *pepck* | Fwd | TGGAAGGCAATGCTCAGCTGT |
|  | Rev | TGCTTTCGATCCTGGCCACAT |
| *p53* | Fwd | AGACTGGATGACTGCCATGGA |
|  | Rev | GCTTCACTTGGGCCTTCAAA |
| *mao-a* | Fwd | TGGCAAGCAAGACATGCTGA |
|  | Rev | ACAAGAACCACAGGGCAGAT |
| *ucp1* | Fwd | AGCTTTGCCTCACTCAGGAT |
|  | Rev | TGGGCTTGCATTCTGACCTT |
| *hmbs* | Fwd | TTCGCTGCATTGCTGAAAGGG |
|  | Rev | TTGCTGAACAGGGACCTGGAT |
| *gapdh* | Fwd | ACTTGAAGGGTGGAGCCAAA |
|  | Rev | GCCCTTCCACAATGCCAAAG |
| *b2m* | Fwd | CATGGCTCGCTCGGTGAC |
|  | Rev | CAGTTCAGTATGTTCGGCTTCC |
| *hprt* | Fwd | TGACACTGGCAAAACAATGCA |
|  | Rev | GGTCCTTTTCACCAGCAAGCT |
